# Primary large B-cell lymphoma of the central nervous system misdiagnosed as autoimmune encephalitis: a case report

**DOI:** 10.3389/fonc.2024.1465961

**Published:** 2025-01-06

**Authors:** Aihui Ren, Guanwen Zeng, Riling Chen, Zhixing Cao, Wenyan Zhuo, Yubin Liang

**Affiliations:** ^1^ Department of Neurology, Zhuhai Clinical Medical College of Jinan University (Zhuhai People’s Hospital), Zhuhai, China; ^2^ Department of ^2nd^Brain Center and Stroke Center, The Affiliated Panyu Central Hospital, Guangzhou Medical University, Guangzhou, China; ^3^ Geriatric Medicine Institute of Panyu District, The Affiliated Panyu Central Hospital, Guangzhou Medical University, Guangzhou, China

**Keywords:** CNS, lymphoma, autoimmune encephalitis, misdiagnosis, case report

## Abstract

Primary central nervous system lymphomas (PCNSL) are rare, constituting 2 - 3% of intracranial malignancies. A 49-year-old male presented with a 20-day history of dizziness and a 15-day history of right-sided weakness. Physical examination revealed various abnormal signs. Initial cerebrospinal fluid (CSF) analysis was unremarkable, while MRI scans (both plain and contrast-enhanced) showed abnormal signals in the left brainstem, thalamus, and basal ganglia regions, with specific enhancement patterns, and arterial spin labeling (ASL) demonstrated hyperperfusion. The patient was initially diagnosed with autoimmune encephalitis (AE) and treated with methylprednisolone with dose reduction and subsequent discharge. However, two months later, his condition deteriorated. Re-evaluation of MRI data, along with magnetic resonance spectroscopy (MRS) results, suggested a neoplastic process. A stereotactic brain biopsy led to a PCNSL diagnosis. The patient was then transferred for high-dose methotrexate chemotherapy but due to lack of regular follow-up, the disease progressed, resulting in cerebral herniation and respiratory failure and ultimately death. The coexistence of PCNSL and AE is diagnostically difficult because of atypical clinical features and non-specific imaging. Thus, for patients with suspected CNS immune-mediated diseases who relapse after steroid treatment improvement, comprehensive evaluation including CSF examination, MRI, and prompt pathological examination is crucial to consider the possibility of PCNSL.

## Introduction

Primary central nervous system lymphomas (PCNSL) are a rare manifestation within the spectrum of central nervous system (CNS) lymphomas, representing a distinct subtype of non-Hodgkin’s lymphoma (NHL). Characterized by its exclusive involvement of the brain, spinal cord, leptomeninges, or vitreoretinal space, PCNSL typically presents with no evidence of systemic involvement (1). PCNSL comprises approximately 4% of all CNS tumors and accounts for 4%-6% of NHLs. Compared to extracranial lymphomas, PCNSL is associated with a poorer prognosis, with a five-year survival rate of approximately 38.3% and a ten-year survival rate of around 30.5%. The majority of PCNSL patients are elderly and immunocompetent, although a minority may have underlying immunosuppression or infections such as Epstein-Barr virus (EBV) or human immunodeficiency virus (HIV). Over the past four decades, the incidence of PCNSL has exhibited a consistent upward trend, with an annual incidence rate ranging from about 0.4 to 0.5 cases per 100,000 individuals. It is now understood that the incidence increases with age, with a median onset age of 65 years, and peaks at approximately 4 cases per 100,000 individuals among those aged over 70 years (2, 3). Illustrating through a detailed clinical case, this article underscores the diagnostic challenges inherent in discerning PCNSL, particularly amidst prior methylprednisolone therapy, and elucidates the diagnostic trajectory culminating in magnetic resonance imaging (MRI) reassessment and brain biopsy for definitive diagnosis. By illuminating this diagnostic odyssey, the article aims to enhance comprehension of PCNSL and mitigate the risk of misdiagnosis (4).

## Case history

**Figure 1:**
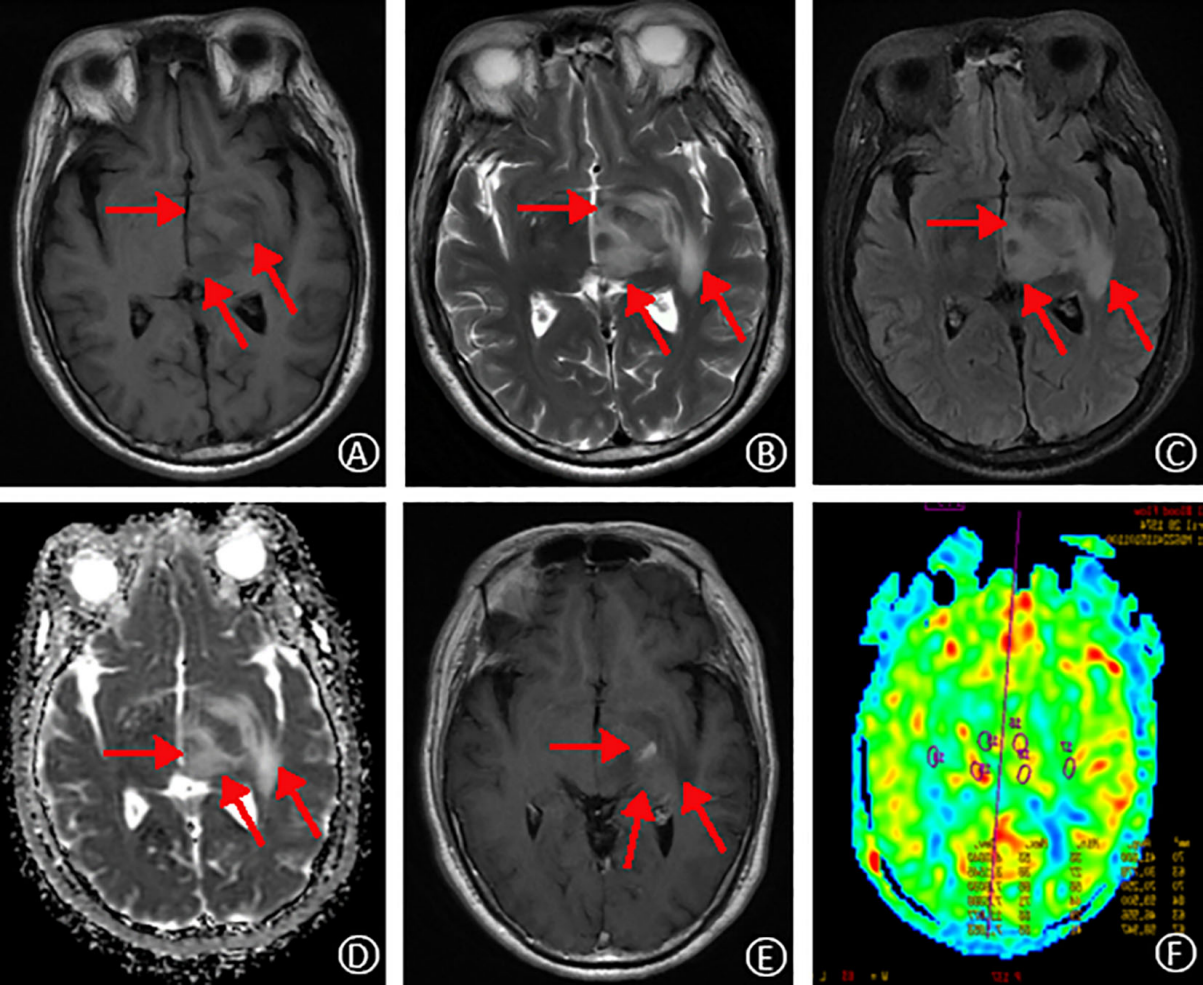
MRI findings on initial admission. T_1_-weighted and T_2_-weighted images, respectively, show slightly prolonged signal intensity in the left temporal lobe, thalamus, and basal ganglia regions **(A, B)**. Fluid-attenuated inversion recovery (FLAIR) sequence reveals high signal intensity in the same regions **(C)**. Apparent diffusion coefficient (ADC) map demonstrates mildly elevated signal intensity **(D)**. Contrast-enhanced T_1_-weighted image shows focal areas of speckled and striated enhancement within the left temporal lobe, thalamus, and basal ganglia **(E)**. Arterial spin labeling (ASL) perfusion map reveals increased perfusion in the corresponding areas of abnormal signal intensity **(F)**.

On October 30, 2019, a 49-year-old male presented to the hospital with a 20-day history of dizziness and a 15-day history of right-sided weakness. He reported the onset of dizziness 20 days prior to admission, accompanied by throbbing headaches and nausea. Additionally, he described progressive weakness in his right limbs over the past 15 days, with a tendency to veer to the right while walking. MRI without contrast revealed hyperintensity in the left midbrain and pons, with abnormal signals in the adjacent left pontine tegmentum, temporal lobe, thalamus, and basal ganglia (**Figure 1**). He was admitted to our neurology department for further evaluation and management. The patient reported a recent weight loss of 3 kg over the past six months, with an otherwise unremarkable medical, personal, marital, and family history. No prior surgical or medical interventions were documented before admission.

A general physical examination revealed a satisfactory condition with no overt abnormalities. Neurological examination demonstrated clear and fluent speech with appropriate responses to questions. Cognitive function was grossly intact with good cooperation during examination. Bilateral pupils were equal, round, and approximately 2.5mm in diameter, with normal light reflexes. Ocular movements were full except for horizontal nystagmus on the lateral gaze to the right. Facial weakness was evident, with a shallow nasolabial fold on the right and a left-sided deviation of the angle of the mouth. Tongue deviation to the right was noted. Cranial nerves III-XII were otherwise intact. Muscle tone was normal bilaterally, but right-sided weakness was present with a grade 4 muscle strength score (using a standardized scale). Finger-to-nose and heel-knee-tibia testing on the right side demonstrated incoordination. Romberg’s sign was positive. Sensory examination revealed no abnormalities. Physiological reflexes were present, and pathological reflexes were absent. Meningeal signs were negative.

Routine laboratory investigations, including complete blood count (CBC), comprehensive metablic panel (CMP), coagulation studies, and HIV, were unremarkable. Similarly, autoimmune markers, such as anti-nuclear antibody (ANA), anti-extractable nuclear antigen antibody (anti-ENA), and double-stranded DNA (ds-DNA) antibodies, were negative. Tumor markers, including carcinoembryonic antigen (CEA), cancer antigen 125 (CA125), and alpha-fetoprotein (AFP), were also within normal limits. Lumbar puncture revealed an elevated cerebrospinal fluid (CSF) pressure of 150 mmHg (reference range: normal). CSF analysis demonstrated a mild pleocytosis with a white blood cell count of 6 × 10^6^/L (reference range: 0-8 × 10^6^/L), predominantly composed of monocytes (66.7%) and lymphocytes (33.3%). The CSF protein level was elevated at 983 mg/L (reference range: 150-450 mg/L). Microbiological studies, including acid-fast bacilli (AFB) stain, India ink stain, and bacterial and fungal cultures, were negative. Additionally, serum and CSF autoimmune encephalitis antibody panels, paraneoplastic neurologic syndrome screening, and CSF metagenomic next-generation sequencing (mNGS) were unremarkable. Contrast-enhanced MRI revealed abnormal signal enhancement in the left brainstem, thalamus, and basal ganglia regions, with a characteristic speckled and striated pattern. Arterial spin labeling (ASL) demonstrated hyperperfusion in the corresponding left brainstem and thalamus. Given the constellation of clinical and laboratory findings, inflammatory lesions remained a possible initial diagnosis.

Correlating the clinical presentation, neuroimaging findings, and CSF analysis, with infectious, neoplastic, and cerebrovascular etiologies ruled out, autoimmune encephalitis (AE) emerged as a leading diagnostic consideration.

## Therapeutic intervention

The patient received a course of methylprednisolone with a dose-tapering regimen. The initial dose was 1000mg daily for three days, followed by a stepwise reduction to 500mg (2 days), then 240mg (1 day), and finally 120mg (1 day). Additionally, the treatment plan included therapeutic dehydration for intracranial pressure management, along with calcium and potassium supplementation, stress ulcer prophylaxis, and symptomatic treatments for neurological deficits and other manifestations. These comprehensive measures led to a significant improvement in symptoms, allowing for discharge from the hospital.

## Follow-up and outcomes

**Figure 2:**
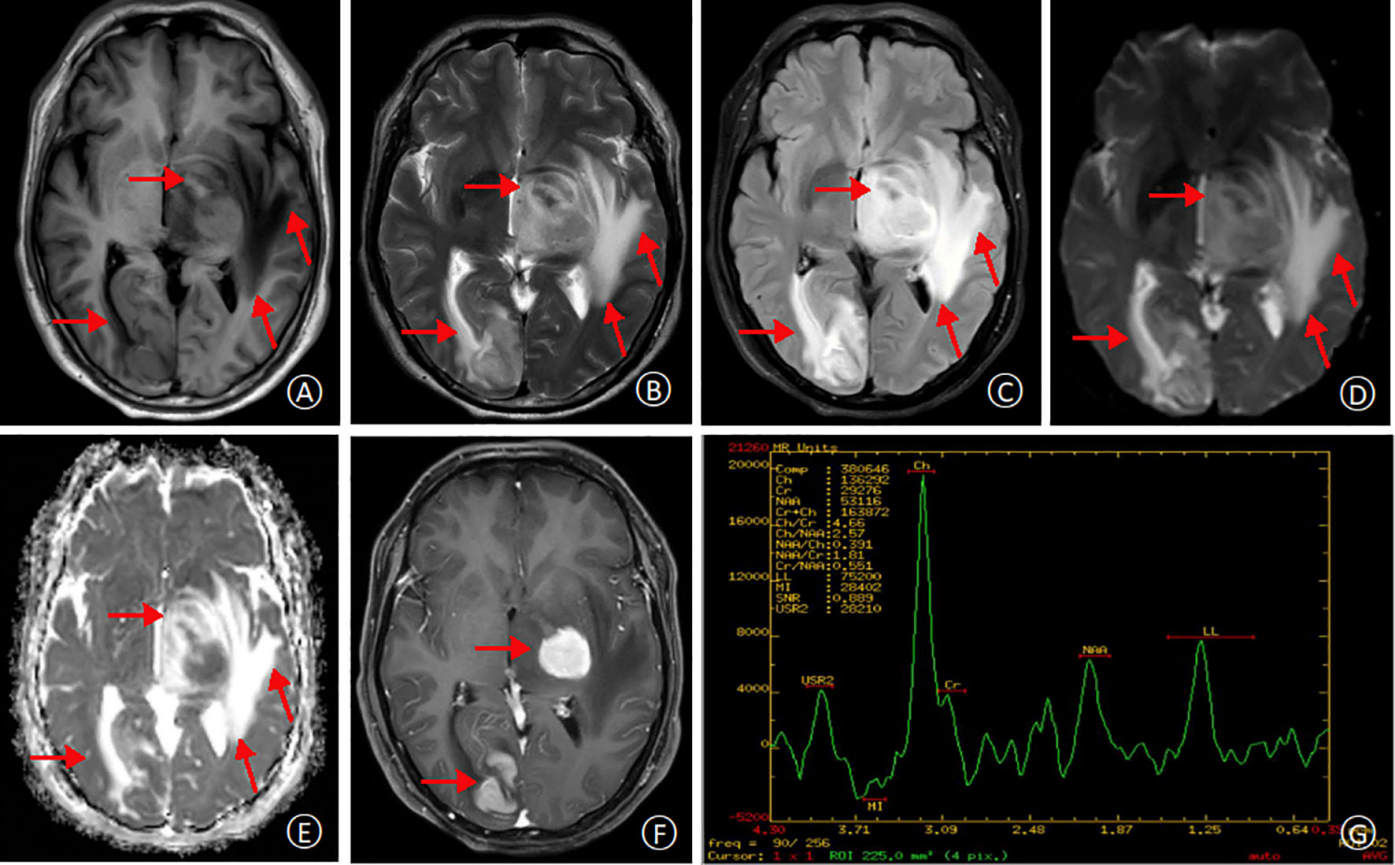
MRI at second admission. T_1_-weighted and T_2_-weighted images, respectively, demonstrating multifocal hyperintensities within the left temporal lobe, thalamus, and basal ganglia regions. The lesions appear larger compared to the previous MRI **(A, B)**. Fluid-attenuated inversion recovery (FLAIR) and diffusion-weighted imaging (DWI) sequences reveal high signal intensities in the lesions, with surrounding areas exhibiting extensive T_1_ and T_2_ hyperintensity **(C, D)**. Apparent diffusion coefficient (ADC) map shows restricted diffusion within the lesions, indicated by high signal intensity **(E)**. Contrast-enhanced T_1_-weighted image demonstrates significant enhancement of the lesions, with a possible “umbilical notch sign”(arrow) **(F)**. Magnetic resonance spectroscopy (MRS) reveals a significant decrease in N-acetyl aspartate (NAA) peak and a corresponding increase in choline (Cho) peak within the lesion area, suggestive of a neoplastic process **(G)**.

**Figure 3:**
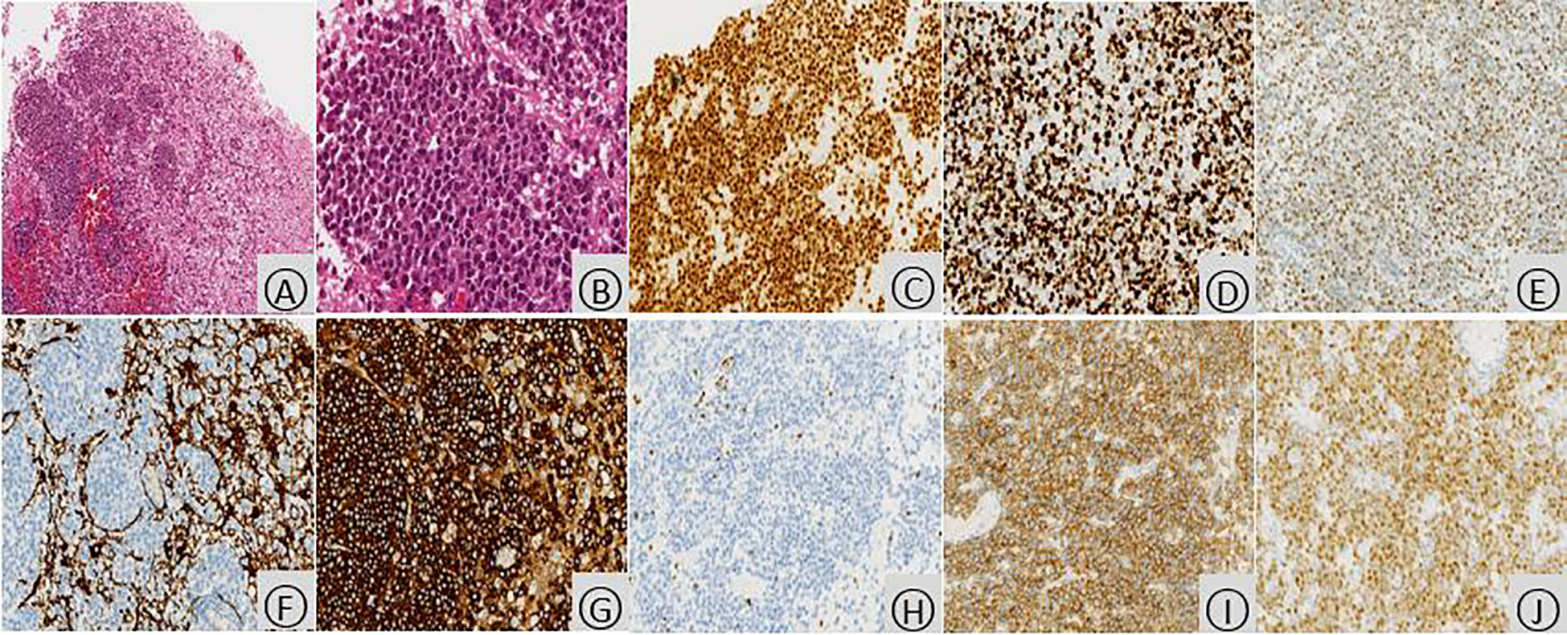
Histopathologic examination of the occipital lobe with PCNSL. Microscopic examination (hematoxylin and eosin stain) revealed infiltration of brain tissue by tumor cells (**A**, ×100). These cells displayed enlarged nuclei with prominent nucleoli, scant cytoplasm, and a relatively uniform appearance. The nuclei exhibited a deep staining pattern with coarse chromatin and readily identifiable mitotic figures (**B**,×400). Immunohistochemical analysis demonstrated diffuse positivity for PAX5 (**C**, ×200), indicating B-cell lineage. The proliferation rate, as assessed by Ki-67 staining, was high, with approximately 80% of tumor cells expressing this marker (**D**, ×200). BCL6, a protein associated with non-germinal center B-cells, was predominantly expressed by the tumor cells (**E**, ×200). GFAP, a marker for glial cells, was negative within the tumor cells, while positive staining was observed in the surrounding residual brain tissue (**F**, ×200). Additionally, the tumor cells were strongly positive for CD20 (B-cell marker) (**G**, ×200), negative for CD3 (T-cell marker) (**H**, ×200), diffusely positive for CD79a (B-cell marker) (**I**, ×200), and mostly positive for MUM1 (another B-cell marker) (**J**, ×200).

Following discharge, the patient’s methylprednisolone dose was gradually tapered. However, during this process, he experienced a recurrence of right-sided hemiparesis (weakness and numbness) accompanied by dizziness and headache. A follow-up MRI scan with and without contrast enhancement was performed on January 16, 2020. The scan revealed multiple new areas of abnormal signal intensity within the left basal ganglia, thalamus, brainstem, right temporal lobe, and right occipital lobe. Compared to the prior MRI from November 3rd, 2019, the lesions demonstrated significant enlargement and increased enhancement. The findings suggested a neoplastic process, possibly lymphoma, although inflammatory granulomatous lesions could not be entirely ruled out (**Figure 2**). Magnetic resonance spectroscopy (MRS) further supported the possibility of a neoplastic lesion, likely a large B-cell lymphoma (**Figure 2**). Given the high suspicion of a CNS tumor, the patient was transferred to our hospital’s neurosurgery department for a stereotactic brain biopsy. The procedure revealed intraoperative findings of grayish-brown brain tissue with solid and soft consistency, localized necrosis, and poorly defined margins. Pathological examination of the right occipital lobe lesion (**Figure 3**) demonstrated clusters of small to medium-sized round tumor cells with prominent nuclei within the brain tissue, exhibiting a focal perivascular growth pattern, Immunohistochemistry analysis revealed the following profile: CD20 (diffuse+), CD79a (diffuse+), Pax-5 (+), MUM1 (diffuse+), BCL-2 (weakly positive in some cells), BCL-6 (mostly positive), CD10 (-), CD3 (-), CD5 (-), CD23 (-), CD138 (-), ALK (-), CD15 (-), CD30 (-), CyclinD1(-), MYC(+), Ki-67(high expression, approximately 80%+), Vimentin (+), EMA (-), CK-pan (-), CgA (-), SYN (-), CD56 (-), SMA (-), Desmin (-), MyOD1 (-), Myoglobin (paranuclear punctate+). HMB45 (-), Melan-A (-), S100 (-), GFAP (-), NeuN (-), Nestin (-), IDH-1 (-), oligo-2 (-), PR (-), CD99 (-). Based on the comprehensive immunohistochemical profile, the definitive pathological diagnosis was diffuse large B-cell lymphoma, non-germinal center type (DLBCL, NOS, non-GCB). Following confirmation of the diagnosis, the patient underwent a right occipital lobectomy in the neurosurgery department. Subsequently, he was transferred to our hospital’s hematology department for bone marrow aspiration. Bone marrow cytology (smear analysis) revealed active marrow proliferation with numerous megakaryocytes and readily identifiable platelets. No evidence of lymphoma cells was observed, although occasional phagocytosis was noted. On February 5, 2020, the patient commenced chemotherapy with a regimen of methotrexate 5.5g on day 1, followed by temozolomide 250mg daily for days 1-5 (cycle 1). This treatment resulted in a significant improvement in symptoms, allowing for discharge home on February 18th, 2020. Unfortunately, the patient did not return for follow-up appointments after discharge. His condition deteriorated rapidly on February 25th, and he passed away on February 28th due to cerebral herniation with respiratory failure. The entire course of the illness spanned approximately four months.

## Discussion

A 49-year-old male had symptoms like dizziness and right-sided weakness. Initially diagnosed with autoimmune encephalitis (AE) and treated with methylprednisolone, he improved and was discharged. But his condition worsened two months later. Further examinations, including MRI and spectroscopy, led to a diagnosis of primary central nervous system lymphoma (PCNSL). He then received chemotherapy in the hematology department. Due to lack of regular follow-up, he died from cerebral herniation and respiratory failure. The coexistence of PCNSL and AE makes diagnosis challenging and misdiagnosis likely. Therefore, comprehensive evaluation is needed for similar patients to exclude PCNSL.

PCNSL is a rare lymphoma subtype, predominantly arising from B-cells, with T-cell involvement being less frequent (5). Common clinical features include headaches, cognitive impairment, neurological dysfunction, and visual disturbances. Diagnosis of PCNSL often relies on a combination of imaging studies, such as MRI or chest computed tomography (CT) scans, to identify the tumor, and CSF analysis to aid in tumor characterization. However, tissue biopsy remains the gold standard for definitive diagnosis (6). Despite advances in therapeutic strategies for PCNSL, the prognosis remains guarded, particularly in elderly patients. Timely diagnosis and intervention are crucial factors in improving patient survival. Unfortunately, in this case, the patient was initially misdiagnosed with autoimmune encephalitis during the course of the illness. This case report highlights the importance of a comprehensive diagnostic workup and the potential consequence of misdiagnosis. By presenting this detailed account and analyzing the reasons for the initial misdiagnosis, we aim to provide valuable clinical insights into the diagnosis and management of similar conditions.

This patient’s initial clinical presentation deviated from the typical course of PCNSL. They presented with dizziness, headaches, and right-sided weakness, which are not specific to PCNSL. Therefore, a broad differential diagnosis encompassing various etiologies was necessary. Lesions associated with AE often involve the limbic system and cerebral cortex, and the clinical picture can be diverse, encompassing dizziness, headaches, nausea, vomiting, cognitive impairment, altered mental status, and focal neurological deficits (7). Conversely, PCNSL typically affects the cerebral hemispheres, thalamus, basal ganglia, corpus callosum, periventricular regions, and cerebellum. The clinical presentation of PCNSL is often characterized by non-specific symptoms such as headache, hemiplegia, sensory disturbances, speech disorders, ataxia, and memory decline (1). Given the significant overlap and atypical presentations in both conditions, a comprehensive diagnostic workup is essential for an accurate diagnosis.

Initial MRI of the patient in early disease had non-specific findings. Flaky, isointense signals were in left brainstem, etc., with speckled contrast patterns in midbrain and thalamus (**Figure 1**). Such can be in autoimmune encephalitis (AE), where typical cases show multiple intracranial lesions, abnormal DWI signals, and may enhance post-contrast (8). PCNSL, however, often has lesions in cerebral areas, with indistinct borders, unique signal traits on T1WI, T2WI, etc. MRS shows lactate and Cho peaks up, NAA down. ASL has reduced blood flow usually, but 20% hyperperfuse. PET/CT shows FDG hypermetabolism (9–11). This patient’s initial MRI differed from classic PCNSL, perhaps due to early steroid therapy. ASL’s high perfusion in left brainstem/thalamus lessened PCNSL odds (12). After high-dose steroids, initial lesions seemed gone, risking misdiagnosis. But with recurrence, follow-up MRI found nodules in multiple brain parts, long T1/T2, high T2-FLAIR and DWI signals, “umbilical notch sign” and midline shift. Frontal-parietal cortices had signals too. MRS suggesting a neoplastic process pointed to lymphoma. For atypical imaging and post-treatment recurrence, review MRI and do MRS promptly to correct diagnosis and avoid errors.

Lumbar puncture findings in autoimmune encephalitis often lack specificity, with normal or elevated intracranial pressure, protein levels, and cell counts. Additionally, CSF analysis may reveal elevated levels of inflammatory mediators like interleukin-6 (IL-6) and tumor necrosis factor-alpha (TNF-α), along with the presence of antibodies specific for various AE subtypes (13). In this case, the patient’s lumbar puncture demonstrated normal intracranial pressure but elevated nucleated cell count and protein levels, with other markers remaining within the normal range. While these findings suggest AE as a possible diagnosis to be ruled out, the ineffectiveness of hormonal therapy and the presence of atypical MRI findings raise suspicions of lymphoma. It is noteworthy, however, that not all AE patients exhibit these distinctive CSF findings, and a negative lumbar puncture does not definitively exclude AE. PCNSL can also present with similar CSF characteristics. Research has shown that CSF analysis, encompassing cytology, immunocytochemistry, and flow cytometry, plays a crucial role in diagnosing PCNSL (14). Sun et al. reported elevated CSF interleukin-10 (IL-10) levels as a potential strong indicator for PCNSL (15). When routine CSF analysis lacks specificity, additional CSF cytology & biomarker analyses are crucial for diagnosis as they’re less affected by other factors.

The patient’s brain tissue pathology showed tumor cell proliferation & infiltration. Immunohistochem was positive for CD20, CD79a etc., diagnosing cerebral lymphoma. Autoimmune encephalitis has inflammatory cell infiltration, neuronal damage, and may show autoantibodies like anti-NMDAR or anti-VGKC, lacking tumor cells (16). PCNSL, on the other hand, typically displays tumor cell proliferation and infiltration within brain tissue with a relatively diminished inflammatory response. Immunohistochemical analysis often reveals the presence of B-cell markers such as CD20, CD79a, Pax-5, and BCL-6 (12). Due to the patient’s atypical symptoms, imaging, and unimproved condition after therapies, a stereotactic brain biopsy was early recommended. This minimally invasive way gets tissue for pathology, key for confirming diagnosis, guiding treatment, and affecting prognosis.

Both PCNSL and AE can demonstrate favorable responses to steroid hormone therapy. This initial response by the patient following high-dose methylprednisolone therapy may have obscured the diagnosis (17). Steroid hormones improve the prognosis of patients with AE by modulating the immune response (18). Given the patient’s initial clinical presentation, signs, and ancillary tests, AE was considered a potential diagnosis (19). During the early stages of AE, high-dose methylprednisolone therapy can lead to rapid symptom improvement. However, in cases of AE recurrence, while steroids may remain somewhat effective, their efficacy diminishes with each subsequent relapse (20). Although initial treatment with steroids may improve symptoms and even cause some histopathological changes, it often leads to rapid relapse and hormone resistance (21). Due to the unpredictable response of PCNSL to steroid therapy and the potential for delayed diagnosis, it is not the preferred treatment modality. In this case, the patient initially responded favorably to methylprednisolone therapy. However, upon relapse, with minimal improvement following additional methylprednisolone, the likelihood of AE became less likely, prompting a shift in suspicion towards PCNSL as a possible diagnosis. Current treatment guidelines for PCNSL prioritize high-dose methotrexate (HD-MTX) as the first-line therapy for patients with a confirmed diagnosis (6). Intrathecal chemotherapy may be considered if meningeal involvement is confirmed and the response to intravenous HD-MTX is inadequate. Decisions regarding radiotherapy, high-dose chemotherapy with autologous stem cell transplantation (ASCT), or omission of consolidation therapy should be made on a case-by-case basis after careful evaluation.

## Conclusion

Accurate PCNSL diagnosis demands considering clinical, imaging, and CSF test features. When unenhanced MRI is unclear, contrast-enhanced MRI, MRS, and CSF analysis are vital. Early recognition and timely biopsy or repeat are key to avoid misdiagnosis and wrong treatment, enhancing patient outcomes.

## Data Availability

The raw data supporting the conclusions of this article will be made available by the authors, without undue reservation.
